# Key influences over actors’ preferences and use of evidence in policy development: insights from the Nigerian IMNCH strategy

**DOI:** 10.1186/1472-6963-14-S2-P76

**Published:** 2014-07-07

**Authors:** Chinyere Mbachu, Ifeanyi Chikezie, Sloudo Eze, Obinna Onwujekwe, Benjamin Uzochukwu, Nkoli Ezumah

**Affiliations:** 1 Health Policy Research Group, College of Medicine, University of Nigeria, Enugu, Nigeria; 2 Department of Health Administration and Management, College of Medicine, University of Nigeria, Enugu, Nigeria; 3 Department of Community Medicine, College of Medicine, University of Nigeria, Enugu, Nigeria; 4 Department of Sociology, University of Nigeria, Nsukka, Nigeria

## Background

Evidence based policy-making has been promoted as a means of ensuring better outcomes but what counts as evidence in policy-making lies within a spectrum of expert knowledge through scientifically generated information. Different actors provide varying degrees of support for and use of different types of evidence in policy development. Since not all forms of evidence share an equal validity or weighting for policy-makers, it is important to understand the key factors that influence their choice of evidence.

## Materials and methods

A retrospective cross-sectional study was carried out at the national level in Nigeria. A case-study approach was used and the Nigerian Integrated Maternal Newborn and Child Health (IMNCH) strategy was selected because it met the criteria of being: (i) recently developed (<10 years old); and (ii) of international prominence. Two frameworks were used for conceptualization and data analysis namely: (i) framework for analyzing the role of evidence in policy making developed by Mirzoev et al., in 2012, and (ii) the policy triangle. They were used to explore the key contextual and participatory influences on choice of evidence in developing the IMNCH strategy. Data was collected through review of relevant national documents and in-depth interviews of purposefully selected key policy and decision makers. Thematic analysis was applied.

## Results

The breadth of evidence used was wide, ranging from expert opinions to systematic reviews. The choice of different types of evidence was found to overlap across actor categories. Key influences over actors’ choice of evidence were: (i) perceived robustness of evidence - comprehensive, representative, recent, scientifically sound; (ii) roles in evidence process, i.e. their degree and level of participation in evidence generation and dissemination, vis-à-vis their role in the policy process; and (iii) contextual factors such as global agenda and influence, timeline for strategy development, availability of resources for evidence generation, and lessons from previously unsuccessful policies/plans.

## Conclusion

Actors’ choice of evidence in policy making is influenced not only by the characteristics of evidence, but on the roles these actors play in the process, their power to influence the policy, and the context in which evidence is used.

